# Communication skills training for physicians improves health literacy and medical outcomes among patients with hypertension: a randomized controlled trial

**DOI:** 10.1186/s12913-020-4901-8

**Published:** 2020-01-23

**Authors:** Seyedeh Belin Tavakoly Sany, Fatemeh Behzhad, Gordon Ferns, Nooshin Peyman

**Affiliations:** 1 Department of Health Education and Health Promotion, Faculty of Health, Mashhad University of Medical Sciences, Mashhad, Iran; 2 Social Determinants of Health Research Center, Mashhad University of Medical Sciences, Mashhad, Iran; 3 Medical Education and Metabolic Medicine Head, Department of Medical Education Brighton and Sussex Medical School, University of Brighton Falmer Campus, Brighton, BN1 9PH UK

**Keywords:** Hypertension outcome, Health literacy, Physicians’ training, Physician communication skill, Blood pressure

## Abstract

**Background:**

Improving the training of physicians about communication skills and patient health literacy (HL) is a major priority that remains an open question. We aimed to examine the effectiveness of communication skills training for physicians on the hypertension outcomes and the health literacy skills, self-efficacy and medication adherence in patients with uncontrolled blood pressure (BP).

**Methods:**

A randomized, controlled trial method was conducted on 240 hypertensive patients and 35 physicians presenting to healthcare clinics in the Mashhad, Iran, from 2013 to 2014. Using stratified blocking with block sizes of 4 and 6, eligible patients with uncontrolled blood pressure were randomly allocated to the intervention and control groups. Physicians in the intervention group received educational training over 3 sessions of Focus –Group Discussion and 2 workshops. The control group received the routine care. The primary outcome was a reduction in systolic and diastolic BP from baseline to 6 months. The secondary outcome was promoting HL skills in hypertensive patients. Data were analyzed using the regression model and bivariate tests.

**Results:**

After the physician communication training, there was a significant improvement in physicians-patient communication skills, hypertension outcomes, medication adherence, and self-efficacy among the patients being managed by the physicians receiving training, compared to the control group.

**Conclusion:**

The educational intervention leads to better BP control; it may have been sufficient training of physicians change to impact counseling, HL and self-efficacy and adherence. The quality of physician-patient communication is an important modifiable element of medical communication that may influences health outcomes in hypertensive Iranian patients.

**Trial registration:**

Iranian Registry of Clinical Trials (IRCT), IRCT20160710028863N24. Registered April 4, 2018 [retrospectively registered].

## Background

Hypertension is an important chronic disease with a high rate of mortality globally [1, 2], which has affected about 1 billion people, and over 7 million people die from it in the world, annually. According to recent studies, hypertension affects approximately 25–35% of the middle-aged people in Iran [3, 4] which is introduced as the first cause of mortality in Iran [5, 6].

There is growing evidence that developing patient experience is a potential way to improve the clinical health outcomes and quality of life for patients with a chronic condition; in part, this may be due to increasing patient cooperation in the treatment process [7, 8]. Today, patient’s cooperation in the treatment process is not only considered as a certain right but is also known as an international gold standard for providers of medical care services who are expected to achieve it [6, 9]. Thus, patients with chronic diseases need adequate HL skills to control their disease and relevant side effects of disease [10]. Several studies showed that effective physician-patient communication has a strong incentive to improve medical outcomes, patient HL, safety patient adherence and psychosocial support [9, 11].

In the recent decade, health organizations have a focus on using HL-informed provider-patient communication strategies as a “universal precautions” approach. Furthermore, they tried to develop an inter-professional care environment and a collaborative practice, which occur when physician and healthcare providers work with patients from outside their profession and within their own profession. This approach is sensitive to health professional’s education program, and are in the pathway linking medical outcome to medical education interventions [12]. According to these strategies, health professionals can be trained to make better use of their knowledge and skills and they are able to more effectively coordinate health care according to patients’ needs [13], therefore, patients could receive a higher quality of health care.

Poor physician-patient communication has been implicated as the main barrier to improve HL and blood pressure control in the Iranian population [14, 15]. Despite the importance of communication skills training, little experimental research has linked skilled physician communication with patient HL and medical outcomes. Further, the interaction between HL skills development and health education interventions has not been well investigated [9, 16] because it is unclear how/where physicians’ communication skills can influence patient HL skills and medication adherence to control blood pressure targets [17, 18]. Thus, there is an increasing need for assessments of strategies, which improve physician-patient communication quality, patient HL and health outcomes in the medical care context [11, 19].

In this study, the primary objectives were to evaluate the effectiveness of physician communication training on hypertension outcome, level of HL skills, self-efficacy and medication adherence among hypertensive patients. Our hypothesis was that there is a direct association between effective physician communication training and successful therapy for patients with hypertension. It is also postulated that because medical system training can overcome most of the theoretical *complexities* that directly link physician education to successful therapeutic. However, the link between health outcomes and physician-patient communication skills is still unpredictable and thus available data are rare.

## Methods

### Study design

CONSORT guideline was used for conducting this trial. This randomized, controlled clinical trial with two parallel arms recruited 35 physicians and 240 hypertensive patients presenting to primary health care centers in Mashhad, Iran, from September 2013 to August 2014. In Iran, healthcare centers are a public sector to provide primary health care services. This primary health care services are provided free of charge in public facility and run by health care professionals such as physicians, family health experts, nutritionists, midwives, and psychologists.

### Participants

In this study, physicians were eligible for inclusion in the study if they (a) did not attend in the communication skills training, (b) participated as member in the Ministry of Health (MOH) sites, and (c) have experience to provide health care services to at least five patients with blood pressure. Physicians’ ages ranged from 26 to 51 (32.03 ± 9.12); they were residents and they provide primary care services, diagnosis, and treatment for patients in the healthcare centers.

This study involved hypertensive patients in interaction with the study of physicians. Patients were eligible for this work if they (a) have uncontrolled blood pressure; (b) carry a diagnosis of hypertension on at least five previous clinic visits; (c) used at least one antihypertensive medication in the last 3 months, and (d) were age 18 or older. They were excluded if patients (a) did not give informed consent; (b) had suffered mental illnesses and disability.

### Sampling and randomization

Sampling began upon the approval of the ethics committee of Mashhad University of Medical Sciences (IR.MUMS.REC.1392.125) and the registration of the study at the Iranian Registry of Clinical Trials (IRCT20160710028863N24). Data collection and recruitment are conducted based on standard guidelines that are described in detail elsewhere [20].

**Fig. 1:**
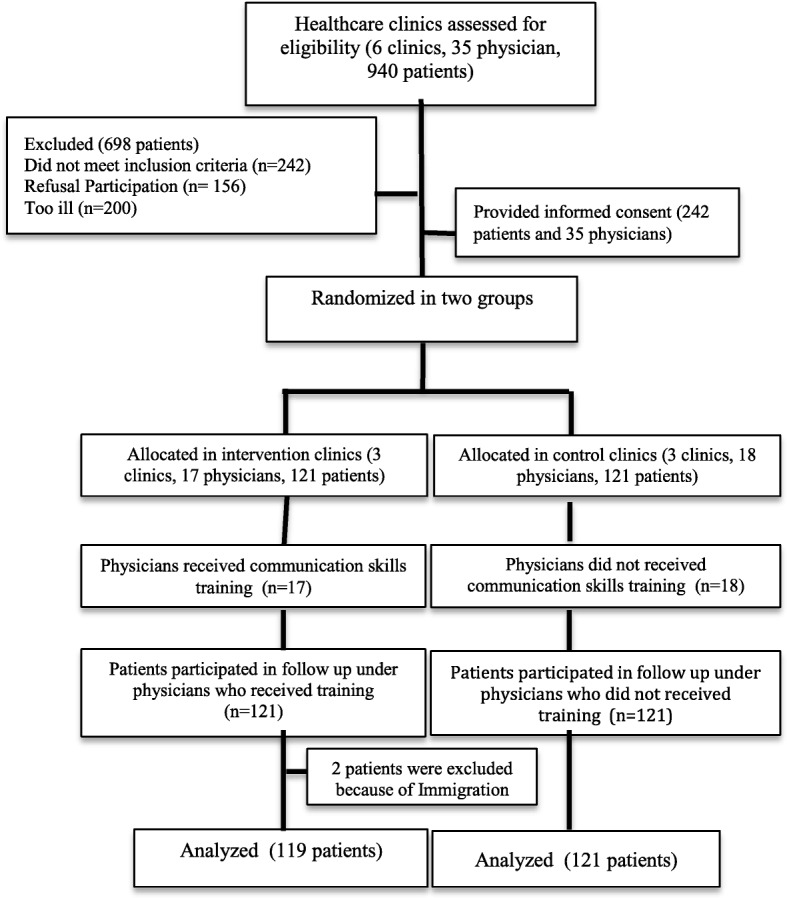
Participants flow chart

In this study, random allocation of healthcare centers was conducted prior to individual recruitment (Fig. 1). Six healthcare centers were randomly selected from a list of centers. Healthcare centers were randomly assigned (as the unit of randomization) to control with usual care (*n* = 3 health care centers) and intervention (*n* = 3) groups. All physicians in 6 healthcare centers were screened, of whom 35 physicians meet the inclusion criteria and they were invited to participate in the study. Then, they completed a Health Literacy Assessment Questions (HLAQs) to assess their communication skills before and after the intervention. The ratio of physicians to patients in his study was 1:7 in each of the clinics. Physicians in the intervention group received educational training, while the control group did not receive educational training.

In this study, 940 patients’ medical records were randomly screened, of whom 242 met the inclusion criteria and 2 participants were lost from follow up. Finally, 240 hypertensive patients were included in the data analysis. These patients were invited to attend in this survey and asked to submit an informed consent form. Eligible patients who had completed the pretest questionnaires were randomly assigned to the intervention (*n* = 119) and control (*n* = 121) groups (Fig. 1) using a computerized table of random numbers and block randomization with block sizes of 4 and 6. The randomization was performed by a member of the study team who was blind to patient assignment and sampling. During the study period, health team members and physicians were aware of allocated groups. Primary and secondary outcomes were assessed by a person not involved in the sampling and group allocation.

After the physicians’ training was conducted, all enrolled patients returned to the health care centers (both control and intervention) for follow-up office visits and asked them to complete a survey. A socio-demographic questionnaire, HLAQs, patient medication adherence, and provider counseling were completed by the patients.

The sample size used for this study was estimated based on power analysis in which a power of 0.8 with a confidence interval of 95% and an alpha of 0.05 for all statistical analyses.

### Data collection tools

The primary outcome in the 6th month included the proportion of patients with controlled BP based on a reduction in systolic and diastolic BP. The secondary outcomes were promoting patients’ health literacy skills, self-efficacy and medication adherence in hypertensive patients.

In this study, we used guidelines for cross-cultural adaptation (CCA) to translate all questionnaires into Persian [20] (Additional file 1: Table S1). The structure intent and content of questionnaires and their relevant items were maintained in the Persian version, which leads to maintaining the original scoring system in the adapted questionnaire. In this study, we used the content and face validity assessment methods to measure the validity of the relevant questionnaires (Additional file 1: Table S1). The reliability of the HLAQs, self-efficacy, and adult primary care were confirmed using a test-retest on 30 people.

The content validity index (CVI) for the HLAQs, self-efficacy, and adult primary care questionnaire scales were 87, 86.2, 89% and content validity ratio (CVR) for these questionnaires were 88, 82 and 91% respectively. The Cronbach’s alpha was estimated as 0.91 for HLAQs, 0.92 for self-efficacy, and 0.93 for adult primary care questionnaire. Likewise, the Intra-class Correlation Coefficient (95% Confidence Interval) was estimated as 0.97 (0.79 to 0.99) for HLAQs, 0.93 (0.82 to 0.95) for self-efficacy, and 0.95 (0.87 to 0.98). A demographic questionnaire, health literacy assessment questions (HLAQs), Chew’s screening questions (CSQ), adult primary care questionnaire, and chronic disease self-efficacy questionnaire were used in this study. The demographic questionnaire, CSQ, adult primary care questionnaire, and chronic disease self-efficacy questionnaire were completed by patients in both groups before (baseline) and 1 and 6 months after completion of the intervention (follow up) through a self-report. To assess providers–patients’ communication skills, the HLAQs was also completed by physicians in both groups before and 1 and 6 months after completion of the intervention.

In this work, uncontrolled BP was considered as an “average systolic BP exceeded 140 mm/Hg or diastolic BP above 90 mm/Hg; or BP above 130/80 for patients with diabetes or renal insufficiency” [20]. Three BP measures were conducted in each medical checkup according to the BP guideline, using an appropriately sized cuff and a calibrated automated BP monitor (Snoqualmie, WA, USA, BPTru device) after 5 min of rest, in the sitting position, with no less than 1 min between measurements. The average of the second and third measures was used [21]. Measurements were completed for eligible patients and physicians in both the control and intervention groups at baseline (before educational training), and 1 and 6 months after completion of the educational training (follow up).

### Physician communication skills

Health Literacy Assessment Questions (HLAQs) were used to examine physician’s communication skills at baseline and follow-up [22, 23]. This is a reliable and valid instrument in health care concepts to assess provider-patient communication skills. This tool includes three sections (spoken, written, and communication skills, patient-provider collaboration and support of patient). This scale contained 33 items with a five -point scale ranging from 0 (poor) to 4 (excellent) and higher scores suggesting physicians have sufficient skills to improve patients’ communication needs, quality of care (e.g., respect and friendliness) and health-literacy limitations, and successfully address them [23].

### Patient health literacy

The Chew’s Screening Questions (CSQ) was used to examine patients’ HL level. This scale has been developed based on the rapid estimate of a test of functional HL in adults (TOFHLA) and adult literacy in medicine (REALM) [24, 25]. This questionnaire includes 4 questions related to a problem learning about medical conditions, confident to fill medical forms and help read hospital material. Responses for all items range from 1 (never) to 5 (always), which combined into a single patient satisfaction composite.

### Medication adherence

We used the Adult Primary Care Questionnaire to examine the effect of physicians’ communication training on improving medication adherence through 29 items [26]. Responses for the 2 first items were bi-optional (1 = no, 2 = yes), while other items were 4-points Likert Scale ranging from 1 (never) to 5 (always). These items asked the patients to assess whether or not physicians guide them to hypertension-related issues in improving medical care and patient concerns about medications [23, 27].

### Patients’ self-efficacy

The Chronic Disease Self-efficacy Questionnaire was implemented to examine patients’ confident related to their regularity tasks or certain activities [28]. This tool included 33 items to examine the following scales: patients’ information about the disease, exercise, communicate with the physician, manage the disease, obtain help from the community, social activities, depression items and manage/control symptoms and shortness of breath. All these items are scored based on five points- Likert from 1 (not at all confident) to 5 (totally confident) [28].

### Intervention

Eligible physicians in the intervention group were invited to receive training, aiming to promote their communication skills with hypertensive patients. The intervention in this study was conducted based on 3 sessions of Focus –Group Discussion (FGD) and 2 workshops (10 h per session).

The FGD sessions, which involved physicians in the intervention group (17 physicians), was conducted by a health educator and cardiologist. In these sessions, physicians expressed their views and experiences of HL improvement in health care clinics with focusing on social problems and patient’s communication needs. In the FGD session, physicians also discussed the existing challenges used to create a successful therapeutic physician-patient relationship. A third session, they concluded that poor clinician support system, patient HL skills and physician communication abilities (medical writing skill, verbal ability, and collaboration) are main barriers to control BP because the physicians do not regularly use HL informed skills in communicating with patients and their families (Table 1).

**Table 1:** Data Analysis Evaluation Plan for Intervention

Process	Objectives	Procedures	Material
FGDs	First Session: Focusing on primary care and communication needs	-Identification of problem and causes of low physicians-patient communication skills -Elicits beliefs, values, about problem -Where does the issue of health literacy come into play? -Why should anyone care if low health literacy exists?	- Didactic presentations - Open-ended questions
	Second Session: Developing focused questions to solve problems Tired Sessions Identifying the targets for intervention	-How does clinical support system information improve problem? -How does support of physician impact patients communication skills? - Discuss available therapies, services - What frustrations do physicians experience with their patients? -How much effort are physician willing to solve the low-health literacy problem? -To identify potential communication gaps to resolve differences of opinion -Offer of help, support of doctor -Offer of improve providers-patient communication skill and clinical support system.	
	First Session: Identifying the targets for intervention	-To identify potential communication gaps to resolve differences of opinion -Offer of help, support of doctor -Offer of improve providers-patient communication skill and clinical support system.	
Educational Intervention Providers is taught based on key communication strategies of HLP model to increase patients’ understanding			
First Workshop (10 h)	Spoken Communication	- The teach-back method, tips for communicating clearly, medication reviews, language differences, and culture and other considerations - How to talk with patients (e,g. does not use medical jargon and talk too fast) - How to used audio, visual aids/video materials to improve their communication with patients (e.g., model of body part, instructional health videos, food models for portion sizes) - How to ask questions by using simple and friendly words	-Teach-back video, -Follow-up Instruction Form, -MedCard, -Brown-bag medication - Review poster
	Written Communication	-Designing easy-to-read tools, -Using health-education signs effectively - Creating a welcoming atmosphere with written materials knows -How to simplify u80and identify materials to easier read? -Consultant assesses educational materials and client forms for readability	-Release of medical information -Lab-results letter -Helpful posters -Educational materials
Second Workshop (10 h)	Collaboration skill To improve self- care behavior and medication adherence	- How to encourage their patients get involved with their care and ask questions - Develop action plans to change patient’s behavior and discuss health care priorities with them -How to teach their patients about taking medications correctly (pill chart and box), offers patients assistance setting up a system, and reviews medications with patients (by health physician) -How to teach their patients about self-management skills (e.g., using a inhaler or glucometer, exercise activity, and dietary advice) -Contacts with patients to understand or to follow up on plans made during the visit.• Getting patient feedback	-Teach-back video PowerPoint - Ask Me poster - Med card - Pill chart - Didactic presentations
	Primary care support To develop physician counseling skills and patients self-efficacy	-Assist patients in overcoming barriers to pharmacologic treatment and to understand their insurance forms and medical bills - Assesses patient’s non-medical barriers to provide appropriate referrals - Support the patient in the use of community-based programs (e.g., weight loss, health literacy, and stop smoking)	-Medical progress notes -Role play exercises -Didactic presentations
Post test (1–6 months follow up)	To evaluate effect of educational intervention	- To assess communication skills between patient and physicians, patients’ self- efficacy and medication adherence, hypertension outcomes, and patient HL	-^a^HLAQs, CSQ, Adult Primary Care, self-efficacy scales and measurement diastolic, systolic

^a^Chew’s Screening Questions (CSQ): Health Literacy Assessment Questions (HLAQs)

Two full-day workshops were conducted by a cardiologist in two consecutive weeks to improve communication skills between physicians and their patients. These workshops were designed based on the Health Literacy in Practice (HLP) model and standard treatment algorithms for hypertension care and management based on the Seventh Report of the Joint National Committee on Prevention, Detection, Evaluation, and Treatment of High Blood Pressure Guidelines (JNC 7). HLP is a facilitated method to increase HL skills, medication adherence and patients’ self-efficacy among patients with chronic disease. Based on this method, physicians must first understand their patients’ HL limitations and communication needs to effectively communicate with them. Likewise, this method emphasizes on patients’ understanding of the self-management abilities and patients’ concerns (side effect and medications) in order to teach them correct methods to take medications [22, 23].

In the intervention group, the physician’s training was conducted using a self-assessment checklist and training package. The main target of these workshops was enhancing the physicians’ counseling skills that affect the behavior change in hypertensive patients. All physicians were trained to: help the patient in overcoming barriers to hypertension treatment, improve engagement with patients, advice the patient about specific health behavior, detect the patient’s sources in changing this behavior, and improve patients’ HL skills to take higher responsibility for their own self-care behaviors (Table 1).

### Data analyses

In the present study, the bivariate analyses (t-test, chi-square, ANOVA) were used to compare clinical and socio-demographic characteristics between experimental and control groups. The linear regression testing the effects of selected covariates (physition-patients’ communication skill) on the dependent variable (pations’ skills, medication adherence, self-efficacy, diastolic blood pressure (DBP) and systolic blood pressure (SBP) scores). The random-effects least squares regression model was conducted to cluster patients within the physician. This model included the main effects of study arm assignment (control vs. intervention), time period (baseline vs. follow-up), and their interaction. Statistical Package for Social Sciences software (SPSS 16, Chicago, Illinois) and R version 3.0.2 were used to produce accurate estimates.

## Results

### Physician and patients’ characteristics

In this study, the majority of eligible physicians were female (64%), had a mean age of 37.08 ± 7.52 years with more than 8 years’ experience to provide primary health care. The majority of hypertensive patients were female (77.34%), married (82.3%), had a mean age of 54.8 ± 11.5, less than diploma degree (42.8%) and employed (86.7%) with an income of 100–200$ monthly (52.1%). Clinically, a high percentage of patients had diabetes (43.6%) and some patients were diagnosed with other comorbid conditions (Table 2)**.**

**Table 2:** Patients sociodemographic and clinical characteristics

Characteristics	Total *n* = 240 (%)	Experimental *n* = 121 (%)	Control *n* = 119 (%)	*P-value*^*a*^
Mean Age (±SD) range: 22–89 years	54.8 ± 11.5	53.8 ± 8.4	54.1 ± 10.1	0.81
Gender				0.701
Female	77.34%	78.2	80.2	
Male	22.66%	21.8	19.8	
Marital status				0.326
Married	82.3%	81.0	85.7	
Widows	11.7%	11.6	10.2	
Divorced	6.26%	8.4	4.1	
Education				0.326
Illiterate	37.5%	40.5	42.0	
< High school	42.8%	38.0	43.7	
> High school	19.7%	21.5	14.3	
Employment				0.745
Employed	86.7	89.1	86.8	
unemployed	13.3	10.9	13.2	
Income per month				0.245
< $100	26.7	25.6	27.7	
100–200$	52.1	48.8	55.5	
> $200	21.2	25.6	16.8	
Comorbid conditions				0.89
Congestive heart failure	3.9	3.4	3.6	
Diabetes	43.6	52.6	46.7	0.26
Coronary artery disease	12.5	12.3	13.9	0.69
Nicotine dependence	3.5	2.9	3.2	0.91
Cerebrovascular disease	6.7	5.7	9.2	0.21
Health literacy				0.321
Inadequate	55.3	55.15	48.87	
Borderline	14.7	10.9	14.5	
Adequate	30	27.4	25.3	
Physician-Patient communication skills				
Limited	66.7	68.4	70.2	0.710
Adequate	33.3	35.6	26.7	
Self-efficacy				
Good	1.2	1.2	1.2	0.52
Moderate	40.8	38.2	43.4	0.24
Poor	58	60.6	55.4	0.31
Medication adherence				
Acceptable	12.4	13.6	11.2	0.31
Moderate	20.6	21.1	20.1	0.45
Poor	67	69.4	64.6	0.42

*Significant at the *p* < 0.05 level, ^a^Testing significant differences between control and experimental groups; ^**b**^ using HLAQs for patients with BP, comprised of 33 items scored on a 4 point scale, higher scores indicate physicians are proficient to improve patients’ communication needs and health-literacy limitations

At baseline, 70% of hypertensive patients showed inadequate and marginal levels of HL (Chew)**.** The results of the physician-patient communication skills showed that 66.7% of the patients needed more support from the physician and they had difficulty in written and spoken communication. Self-efficacy was poor (58%; *n* = 139) or moderate (40.8%; *n* = 98) for most patients and good for only 1.2% (*n* = 3). The level of adult primary care questionnaire was low (160 patients; 67%) for most of the patients, while it was temporarily acceptable for 12.4% (30 patients) (Table 2).

Based on our results, patients with adequate HL were significantly showed better medication adherence (*ß* = 0.19, *p* = 0.001), self-efficacy (*ß* = 0.14, *p* = 0.023), DBP (ß = 0.13, *p* = 0.032) and SBP (ß = 0.12, *p* = 0.03) scores than patients who have lower level of HL and communication skills. Similarly, educational attainment was significantly associated with patients’ HL (*ß* = 0.15, *p* = 0.02) (Additional file 1: Table S2)**.**

### Educational intervention

A significant difference (*p* < 0.05) was found between participants (physicians and patients) of intervention versus control groups at follow-up, and in change from baseline to follow-up in all scores including physician’s communication skills, patient’s HL skills, medication adherence, patients’ self-efficacy, SBP and DBP (Table 3)**.** Random effects least squares regressions models also showed the significant evidence in the change in physician’s communication skills (CI: 0.27, 0.91, P: 0.00), patient’s HL (CI: 0.22, 0.83, P: 0.00), medication adherence (CI: 0.20, 0.67, P: 0.00), patients’ self-efficacy (CI: 0.14, 0.65, P: 0.002), SBP (CI: − 1.02, − 0.31; P: 0.00) or DBP (CI: − 1.32, − 0.49: P: 0.00), from baseline to follow-up, in the intervention compared to the control group (Table 4)**.**

**Table 3:** Average scores for each outcome from baseline to follow-up

Variables	Baseline			Follow up		Variation from baseline to follow up^*^			
	Intervention	Control	*P-value*	Intervention	Control	*P-value*	Intervention	Control	*P-value*
Physician *(n = 35)*									
Physician-patient communication skills *Spoken communication* *Written communication* *Collaboration* *Patient support*	63.3 ± 12.04	68.7 ± 0.06	0.18	80.33 ± 10.7	69.9 ± 4.4	0.007	17.04 ± 13.2	1.15 ± 5.9	0.001
	20.5 ± 3.34	21.76 ± 2.4	0.29	26.7 ± 2.6	22.03 ± 2.54	0.001	6.16 ± 3.95	0.26 ± 1.72	0.001
	18.95 ± 4.71	21.34 ± 4.89	0.22	24.75 ± 3.22	22.01 ± 3.08	0.04	5.79 ± 6.30	0.65 ± 5.18	0.038
	12.3 ± 3.01	13.65 ± 1.3	0.15	18.6 ± 2.58	13.8 ± 1.23	0.03	5.3 ± 2.85	0.07 ± 0.27	0.001
	11.54 ± 2.38	12 ± 1.63	0.58	15.32 ± 3.5	11.15 ± 2.08	0.014	3.8 ± 2.15	0.15 ± 2.18	0.001
Health outcomes *(n = 240 patient)*									
Patient health literacy	1.84 ± 0.68	1.67 ± 0.43	0.18	2.52 ± 0.8	1.72 ± 0.28	0.031	0.68 ± 0.25	0.35 ± 2.17	0.024
Medication adherence	78.08 ± 13.59	80.38 ± 11.2	0.15	95.42 ± 10.8	80.87 ± 9.44	0.001	17.33 ± 12.9	0.48 ± 9.08	0.001
Patients’ Self-efficacy	120.24 ± 22.7	119.4 ± 21.6	0.75	127.4 ± 17.1	120.9 ± 16.9	0.003	7.14 ± 12.33	1.47 ± 9.77	0.001
SBP	145.6 ± 13.8	146.1 ± 15.2	0.21	124.2 ± 7.2	143.8 ± 13.9	0.024	−21.4 ± 6.1	−2.3 ± 1.03	0.001
DBP	91.50 ± 9.6	89.53 ± 9.6	0.32	78.16 ± 6.3	87.16 ± 10.3	0.14	−13.02 ± 3.5	−2.37 ± 0.7	0.032

±: Showing mean score (standard deviation); *Small discrepancies in variation scores due to rounding;

**Table 4:** Interaction of Intervention with time period for each outcome

Outcome^a^	Estimated Parameter (SE^b^)	95% Confidence Interval (CI)	P-value
Physician			
Physician-patient communication skills	0.61 (0.16)	0.27, 0.91	0.00
Patients			
Patient health literacy	0.59 (0.12)	0.22, 0.83	0.00
Medication adherence	0.43 (0.11)	0.20, 0.67	0.00
Patients’ Self-efficacy	0.41 (0.19)	0.14, 0.65	0.002
SBP	−0.75 (0.18)	−1.02, −0.31	0.00
DBP	−0.93 (0.22)	−1.11, −0.49	0.00

^**a**^Adjusted for age, gender, marital status, employment, income, education, comorbid conditions; ^b^ Standard error

## Discussion

Our findings showed that physician communication training may directly impact hypertensive outcomes (DBP and SBP), and more often it had an indirect effect on hypertensive outcomes through its influence on intervening variables (e.g., patients self-efficacy, patients’ HL skills, and adherence to treatment). As matter of fact, health providers communication skills intervention may do so directly by activating patients to talk about their problem (medication, side effects, social and financial and problem), which prompts the physitions to improve their communication skills, counseling behaviors, clinical support and change medication, which leads to better blood pressure control among patients [29, 30]. This result is in agreement with recent studies that found effective physician-patient communication is the heart of medicine in the delivery of health care [9, 11, 31]. In a sample of diabetic patients, 11% absolute modification in medication was observed in patients who perceived by their physician to be effective communication behavior [32]. Similarly, Schneider et al. in 2004 found that higher level of the physician-patient adherence dialogue about chronic disease treatment, evaluated as the ability to understand problems, the quality of physician’s information-sharing methods, and suggest help with medications, described the main variance between medication adherence and perceived quality of the physician-patient communication [33].

Furthermore, we found that patients with higher education were significantly associated with a high level of HL and better medication adherence, which is in contrast with recent studies in hypertensive patients [29, 30]. These studies indicated that usually patients with lower educational qualifications observed to be less knowledge about medical care, less healthy, to have lower HL skills and more difficulties to understand and read health care information [11, 34, 35].

This study is the first evaluation of communication skills training implemented for physicians, which examine the association between physician-patient communication skills and medical outcomes among hypertensive patients with low HL skills, who at much greater risk for the poor quality of life and medication adherence to treatment than that other populations [6, 23]. This study indicates the short-term experiential physician communication skills training to modify medical outcomes, patient HL for up to 6 months. This intervention program seemed to benefit all health professionals, irrespective of baseline patient experience/HL skills.

Some limitations are worth noting. First, the duration of the medical communications between the physician and patient was not evaluated. The importance of regular primary care provider is well documented in hypertensive patients because lead to significant patient satisfaction with the diagnostic than who do not have a continuity of care [6, 36]. Second, a self-report item was used to determine medication adherence and HL skills, which may overestimation of each score level. Although, we checked overall authenticity responses and we approved that these responses are closely linked to clinical characteristics of patients. Further study is still required to consider the strengths of subjective and objective assessment to detect the relevant compounds that modify physician-patient communication and make actual health outcomes. Likewise, a future investigation would benefit from evaluating patient and physicians characteristics (e.g., race, personality traits, age).

These findings highlighted the urgent need to develop an effective intervention program during routine practice to improve collaboration between patient and physician. Despite the negative implications of low physician-patient communication skills and patient’s health HL on health outcomes, physicians and other health care providers are usually unaware of HL problems in their patients. Therefore, physicians need to be, first, aware of what HL skills is, and how it can compromise caregivers’ ways to modify health outcomes in their patients. Secondly, the physician should be able to identify patients with low HL skills and to plan appropriate educational health education. In addition, the training program must be systematically implemented for graduate medical education to achieve effective collaborative communication by a physician and other health providers. This is important to increase opportunities for medical students to use open discussions and narratives into the curricula because the use of these narratives is more likely to encourage their self-reflection during residency, a value shared decision-making, increase their satisfaction and confidence when using these communication skills during patient interactions.

## Conclusion

This communication training intervention for physicians offers multiple benefits including improved patient HL, medical outcomes, medication adherence, and self-efficacy. Our finding showed that physician with high communication skills with their patients was associated with better HL skills to improve their medical care. Therefore, physician communication skills training appears to be an efficient way to clarify the needs of patients with limited HL and improve communication challenges between patients and their physicians.

## Supplementary information

**Additional file 1 **: **Table S1**. Translation process according to international guidelines for cross-cultural adaptation (CCA) in this study. **Table S2.** Results of linear regression testing the effects of health literacy on health outcomes and demographic characteristic

## Data Availability

Data and materials can be requested from the corresponding author.

## Abbreviations

CCA: Face and content
CSQ: Chew’s Screening Questions
CVI: and content validity index
CVR: The content validity ratio
EHRs: The electronic health records
HL: Health literacy
HLAQs: Health Literacy Assessment Questions
HLP: Health Literacy in Practice
MOH: Ministry of Health
