# Supplementary material for: Communication skills training for physicians improves health literacy and medical outcomes among patients with hypertension: a randomized controlled trial
Source: BMC Health Serv Res. 2020 Jan 23;20:60. doi: 10.1186/s12913-020-4901-8 (PMC6979365; doi:10.1186/s12913-020-4901-8)
Supplement: Supplementary file 1 — Additional file 1 : Table S1. Translation process according to international guidelines for cross-cultural adaptation (CCA) in this study. Table S2. Results of linear regression testing the effects of health literacy on health outcomes and demographic characteristic [file 12913_2020_4901_MOESM1_ESM.docx]

Table S1: Translation process according to international guidelines for cross-cultural adaptation (CCA) in this study

| **Translation steps** | **Description** |
| --- | --- |
| **Forward Translation** | - 3 forward translations were conducted by 3 independent native Iranian translators from English into Persian language.  - Independent translators were fluent in English language and they instructed to use wording that could be understood by a patients  - 2 translators were medical interpreters who aware of the outcomes analyzed by the questionnaires |
| **First Unified Forward Translation** | A meeting was convened among the 3 forward translators and the authors of this study to reach a consensus among the members of the group and to produce a first unified Iranian version of the 3 forward translations. |
| **Backward Translations** | - The first unified Iranian version of the questionnaires was back-translated into English by 3 bilingual professional translators who were fluent into the colloquial and idioms forms of the forward language.  - The back-translators were different persons from the forward translators and had not seen the original text of the questionnaires, were unaware of the target of the research,  - Back-translation was conducted to improve the quality of the final version of a questionnaire, by amplifying any misunderstandings in the forward translations. |
| **Review of Backward Translations.** | - The 3 backward translations were reviewed by the authors (academic staff in health education department) of this paper and by the 3 specialists in cardiovascular disease (expert panel) to check their correspondence with the original version of the respective instruments.  - The aim of this phase was to make sure that all the items and instructions were still relevant based upon the original version and fully comprehensible,  - They verify the cross-cultural equivalence of the source and final versions by comparing their idiomatic, experiential, semantic, and conceptual equivalencies of each items. |
| **Second Unified Forward Version** | - This meeting was convened among all of the translators to discuss about reviewers’ comments related to the backward translations.  - The purpose was to reach a consensus among the translators for a second unified version of the questionnaires |
| **Pre-testing in a Target Population** | - The second unified version was administered to 90 hypertensive patients and 30 health providers to test participant comprehension in the target population, using probe method.  - A health professional asked participant to reflect the clarity, readability and simplicity of instruments.  - The health professional judged whether the question was perfectly understood by each patient or providers. An expert panel reviewed the scale. They assessed the relevance and necessity questions in order to calculate the content validity index (CVI) and content validity ratio (CVR). The relevance of the items was assessed using a four-point rating scale ranging from 1 (=not relevant) to 4 (=very relevant). The necessity of the items was assessed using a three-point rating scale ranging from 1 (=not necessary) to 3 (=essential). |

Table 2S: Results of linear regression testing the effects of health literacy on health outcomes and demographic characteristic

| **Variables *n* = 240** | ***ß (S.E)*** | ***P*** |
| --- | --- | --- |
| Age | 0.02 (0.08) | 0.83 |
| Gender | -0.05 (0.13) | 0.33 |
| Marital status | 0.03 (0.15) | 0.23 |
| Education | 0.15 (0.004) | 0.02 |
| Employment | 0.04 (0.09) | 0.73 |
| Income | -0.04 (0.14) | 0.36 |
| Comorbid conditions | -0.06 (0.04) | 0.21 |
| medication adherence | 0.19 (0.10) | 0.001 |
| Patients’ Self-efficacy | 0.14 (0.002) | 0.023 |
| DBP | 0.13 (0.002) | 0.032 |
| SBP | 0.12 (.004) | 0.03 |
